# Floundering or Flourishing? Early Insights from the Inception of Integrated Care Systems in England

**DOI:** 10.5334/ijic.7738

**Published:** 2024-07-02

**Authors:** Bethan Page, Thavapriya Sugavanam, Ray Fitzpatrick, Helen Hogan, Mirza Lalani

**Affiliations:** 1 Cicely Saunders Institute, King’s College London, London, UK; 2 Department of Experimental Psychology, University of Oxford, Oxford, UK; 3 Nuffield Department of Population Health, University of Oxford, Oxford, UK; 4 Nuffield College, University of Oxford, Oxford, UK; 5 Department of Health Services Research and Policy, London School of Hygiene and Tropical Medicine, London, UK

**Keywords:** Integration, Integrated Care Systems, system reorganisation, population health

## Abstract

**Background:**

In 2022, England embarked on an ambitious and innovative re-organisation to produce an integrated health and care system with a greater focus on improving population health. This study aimed to understand how nascent ICSs are developing and to identify the key challenges and enablers to integration.

**Methods:**

Four ICSs participated in the study between November 2021 and May 2022. Semi-structured interviews with system leaders (n = 67) from health, social and voluntary care as well as representatives of local communities were held. A thematic framework approach supported by Leutz’s five laws of integration framework was used to analyse the data.

**Results:**

The benefits of ICSs include enhancing the delivery of good quality care, improving population health and providing more person-centred care in the community. However, differences between health and social care such as accountability, organisational/professional cultures, risks of duplicating efforts, tensions over funding allocation, issues of data integration and struggles in engaging local communities threaten to hamper integration.

**Conclusions:**

Despite ICS’s investing in the structural and relational components of integrated care, the unprecedented pressures on systems to reduce demand on primary and emergency care tackling elective backlogs may detract from a key goal of ICSs, improving population health and prevention.

## Introduction

Health and care systems globally are facing the unprecedented pressures of increasing needs from an ageing population, rising workload for an overburdened workforce and limited financial resources – issues compounded by the Covid-19 pandemic [1,2]. There is a growing consensus that better integration of care is a key part of the approach to tackling these challenges [3,4]. The term ‘integration’ is used interchangeably but represents a ‘joining up’ of traditional silos of care across (horizontal) and within (vertical) systems, organisations, services and service providers [5]. Several countries have taken steps to integrate care both structurally (aligned governance, financial and managerial arrangements) and relationally (partnership working, changing organisational and professional cultures), moving care away from hospitals into the community allied with a greater focus on prevention and improving population health [6,7].

Despite ongoing reorganisation toward a more integrated system, the evidence base for integration to date indicates a mixed impact on the quality of care. Greater integration through expansion of community services may not result in cost efficiencies [8] or improvements in performance outcomes. For example, there is minimal evidence to suggest that out of hospital community-based initiatives reduce unplanned hospital admissions [9]. Even so, integrated care approaches may improve patient satisfaction and experience of care while also enhancing access to services [10].

### Integrated Care Systems in England: A new macro level innovation

Since the introduction of the 2012 Health and Social Care Act in England there has been significant investment in integrated care initiatives; ‘Vanguard’ programmes to test ‘New Care Models,’ (2014), Sustainability and Transformation Partnership (STP) (2015) and more recently Integrated Care System (ICSs) [11,12]. Each of these developments are innovative – rarely had health and social care organisations come together with the intention of tackling mutual problems at scale. The innovations were underpinned by the premise of transferring care away from hospitals to community settings, as well as increased collaboration between individual institutions and more recently, a focus on improving population health [13]. However, despite the policy imperative for integration, the evidence base for integrated care in an English context is unclear [4]. This can be attributed to the heterogonous approaches to evaluation of such initiatives and the heterogeneity of the local systems being evaluated [11]. Hence, the indeterminate picture of the benefits and outcomes of integrated care has led to some commentators suggesting that the 2012 Health and Social care Act served to catalyse fragmentation of the health service, increasing competition between providers rather than promoting local collaboration, as it was intended to [8]. Indeed, a rapidly changing policy context, a propensity for centralised governance juxtaposed with individual provider priorities, may have hindered the joining up of services [14].

**Figure 1:**
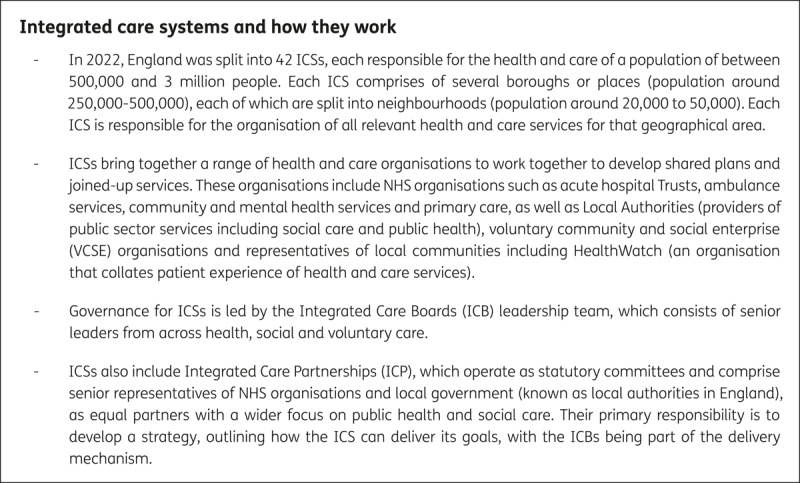
Integrated Care Systems (ICSs) in England and how they work.

Despite such concerns, in 2019, the National Health Service (NHS) Long Term Plan included the aim that the entire country would be covered by around 40 ICSs which would result in a shift away from the previous model of commissioners (through Clinical Commissioning Groups (CCGs)) and providers [15]. ICSs were expected to bring health and care organisations and services together to work more effectively on a broad population-level agenda including prevention, addressing health inequalities, improving care outcomes and better management of resources. Figure 1 describes the key characteristics of ICSs and how they work.

### Understanding integration in practice – Leutz’s five laws of integration

**Figure 2:**
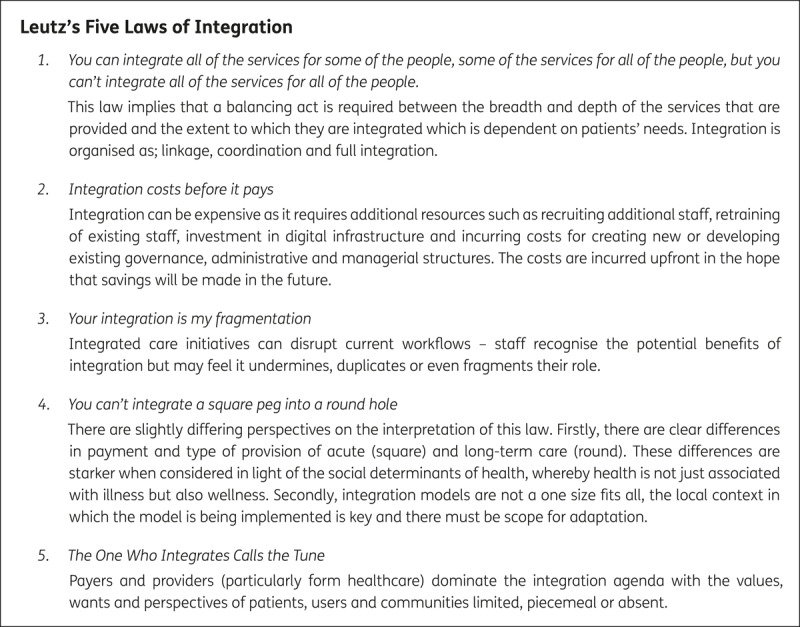
Leutz’s Five Laws of Integration [16].

The challenges for integrated care were highlighted in a prescient paper by *Leutz et al* who reflected on early issues from the emerging shift in both the US and UK in the 1990’s from traditional siloed organisations and sectors of care to more integrated working across health and social care [16]. Leutz posited five laws that illustrated the key factors thought to inhibit (and facilitate) the integration of health and care systems and services in the 1990’s. These are outlined in Figure 2. The laws were designed to highlight the potential challenges of integration to system leaders, managers and professionals cautioning them about the barriers that would need to be overcome to achieve integration while recognising that the knowledge base and experience about integration was still evolving. Furthermore, Leutz made three key recommendation for systems based on the laws; i) incorporate the views of users and communities, ii) integrate, coordinate and link services for persons with disabilities and chronic conditions and iii) clarify the boundaries of care between health and other sectors. Twenty years on, integration efforts in several countries continue to grapple with how best to implement these recommendations [17].

Leutz’s framework supports our understanding of the key challenges of integrating care at every level from micro to system and it was developed at a time when integration as an innovative approach to system reorganisation was still emerging. Hence, it is particularly useful in exploring the early development of nascent and ambitious ICSs in England that are looking to integrate across all levels of the system. In contrast, *Fulop et al’s* six dimensions for effective integration provides a more theoretical perspective on integrated care and therefore may be more pertinent when assessing longer established integrated care systems and initiatives [18]. More recently, *Shortell et al* applied Leutz’s five laws to Accountable Care Organisations (ACO) in the United States concluding that Leutz’s three recommendations outlined above, remain relevant today; i) greater engagement with approaches such as co-production has increased patients, users and community involvement in service provision, ii) shared electronic records, increased availability of digital tools and an emerging role for artificial intelligence and machine learning supports better integration, coordination and linkages between services and iii) extending professional roles and responsibilities, role substitution or task delegation may lead to a greying of boundaries between sectors rather than providing clarity. Whether in the long term, this improves or impedes patient and service outcomes is not clear [19].

This study is situated within a wider research project [20] that aimed to assess how ICSs are managing and improving quality defined as effectiveness, safety and experience [21,22]. Using this framing, ICSs are responsible for achieving the best outcomes for individuals and populations through the provision of evidence-based services to all who can benefit, whilst avoiding harm, and ensuring care is compassionate, dignified and respectful. This study aims to assess the progress of developing English ICSs using *Leutz et al’s* five laws of integration as an organising framework. Progress will be assessed against the various relevant components of each law that influence integration in the context of ICS’s in England. We aim to develop an understanding of how ICSs are developing as nascent organisations as well as the key challenges and enablers to integration.

## Methods

The study was a qualitative case-study of four ICS sites which consisted of semi-structured interviews, meeting observations and documentary analysis.

### Settings and subjects

The four ICS sites were purposively sampled on the basis of geography (urban/rural), demography (population size) and pre-existing system architecture (e.g. history of system-wide working and/or related experiences that support partnership working across health and social care such as the Multi-Community Provider Vanguard programme in England) [23]. The study took place between November 2021–May 2022, a key time period in the development of ICSs ahead of their official launch in July 2022. At the time of our study, ICS were in their infancy, although two ICSs had a longer history of system-wide working as early STP sites – the precursors to ICS [12]. Further information about the four ICS case sites is provided in Table 1.

**Table 1:** Description of the four ICS case sites.


	POPULATION SIZE	RURAL/URBAN	HISTORY OF PARTNERSHIP WORKING

**Site A**	1 million, split into 4 place-based partnerships	Mixture	Formed as an ICS in 2019 (3 years before the ICS became legal entities) History of some existing partnerships between health and care organisations at system level

**Site B**	1.8 million, split into 5 place-based partnerships	Mixture	One of the places was previously awarded Vanguard status (support and funding to develop innovative models of care which other parts of the country can learn from) Limited history of partnership working at system level

**Site C**	1.1 million, split into 5 place-based partnerships	Primarily rural with one major urban centre	Limited history of partnership working at system level

**Site D**	2 million, split into 7 place-based partnerships	Urban	One place was previously a Vanguard site Formed as an STP prior to becoming an ICS, and comprised of three sub-system partnerships Extensive history of partnership working across health and care at sub-system and system level


### Data collection

Interviews were held with senior leaders and other key stakeholders across the four ICS (n = 67) to ascertain key aspects of the early development and subsequent progress of ICSs. In each ICS we worked with the lead for quality (our key contact) to help identify a list of suitable research participants. We purposively sampled a diverse range of actors from across the health, social and voluntary sectors as well as those representing local people and communities to participate in interviews. We also used a snowballing approach to identify participants from a broad range of job roles. This ensured that we garnered a broad range of perspectives on the management and assurance of quality in ICS. We also observed relevant ICS meetings and reviewed ICS specific documents. A comprehensive list of data collected is described in Table 2.

**Table 2:** Overview of research methods and data collection.


RESEARCH METHOD	PARTICIPANTS/MEETINGS/DOCUMENTS

Observation of meetings	Meetings included: Integrated Care Partnership Board Integrated Care Board Clinical Commissioning Group Governing Board Quality forum System Quality Group System Delivery Group ICS CEO group

Interviews (n = 67)	ICS Exec team (n = 13): CEO, Independent Chair, Chief Information Officer, Other Exec members. Senior representatives in NHS acute and community and mental health Trusts (n = 13). CCGs and primary care (n = 7): CEO/deputy CEO, Chief Nurse, Director of Nursing, Clinical Chair, Director of Transformation, Performance and Assurance Quality leads: Chief Nurse, Chief Quality Officer, Lead for Quality Development (n = 13) Local Authority representatives: Director of Adult and Children Social Care Services, Director of Public Health (n = 13) Others: HealthWatch, VCSE and Public Involvement representatives (n = 8)

Documentary analysis	Meeting minutes and documents, ICS strategic plans, ICS quality strategy and framework


The project received ethics approval from the University of Kent (ref LSSJ0459). The researchers approached potential participants by email, outlining the purpose of the study and the interview process. Written informed consent was obtained from each participant prior to the interview. The researchers assured participants of confidentiality and anonymity and explained that participation was voluntary, and that they were free to withdraw from the study. No participants withdrew their consent.

Interviews were guided by a topic guide, focusing on overall ICS perspective on quality, how ICSs are organised to address quality, the internal and external influences on the ICS approach to quality and capacity of ICS to address quality. The topic guide was informed by empirical and theoretical literature, and current policy documents on the set up of ICSs. All interviews were conducted on MS Teams, audio-recorded and transcribed verbatim. Interviews lasted between 45–60 minutes.

#### Data analysis

We conducted qualitative analysis using a thematic framework approach to identify patterns and themes [24]. The qualitative data management tool Nvivo 12.0 was used to manage and code the interview data. A coding framework was developed based on the interview topic guide, the data from observations and documents. The research team met every two weeks during the data collection and analysis phase to discuss emerging themes and sub-themes and to develop the coding framework which was updated iteratively. The research team comprised of nine experienced qualitative researchers, including researchers with experience across healthcare, social care and patient and public involvement: five of these researchers conducted the analysis, with the others provided feedback on the framework and presentation of the results. Field notes from the meeting observations and reviews of document were also used as contextual information to help support the analysis of the interview data. During the analysis of the data, substantive content was identified on issues associated with integrating health and care which have also previously been described elsewhere in evaluations of integrated care initiatives in England [2,11,25,26]. These components of the data were reanalysed and mapped to *Leutz’s* framework by three of these researchers, one with substantial expertise in the reorganisation and integration of health and social care services. Consensus was reached through regular meetings, and consultation with the wider research team.

## Findings

All four of the ICS case sites were experiencing significant challenges in setting up as new organisations responsible for the health and care of their respective populations. Despite their varying population needs and history of partnership working, all sites faced very similar challenges. We map the relevant findings to Leutz’s five laws of integration.

### Law 1: You can integrate all of the services for some of the people, some of the services for all of the people, but you can’t integrate all of the services for all of the people

ICS were initially focussed on infrastructure development to support integration with a disparate range of approaches across the different levels of integration; linkage, coordination and full integration [16].

Work was ongoing to identify key areas of overlapping priorities and interdependencies where joint working would be most beneficial for all health and social care partners. Local authorities preferred to focus on areas of linkage and coordination, rather than full integration e.g. areas of interdependency such as hospital to community discharge of people who require social care support, as this was seen as both an efficient use of resources and pertaining to the routine work of social care.

*“I don’t see the ICS sucking in the oversight of everything that happens in the health and care economy. I think if I was drawing it, I’d draw it as a Venn diagram. … I don’t really care what happens in the orthopaedic part of the hospital because it’s never going to touch my world in terms of social care. I mean, there will be some hip replacements and people that need support on discharge, but generally, young people with orthopaedic things isn’t going to touch the world of social care. Do I want to be involved in that?”* [Director of Adult Social Care Services]

It was thought that integration at place or neighbourhood level rather than system level would enable the ICS goals of improving population health and reducing inequalities to be met. Interviewees gave examples of place level priorities where there could be benefits from working together across partners such as focusing on local populations where there was greater need (including unmet need) such as initiatives to improve the health and wellbeing of children and young adults under local authority care.

A key challenge facing ICSs was how data could be integrated between the various organisations involved in the ICS. Full integration (integrating data across health and social care) particularly to address inequalities was an ambitious goal for some interviewees but it was recognised that it could be hampered by issues of information governance such as data confidentiality and data sharing agreements between the health and care sectors and gaps in data in primary and social care. One example of where data integration was helping was shared care records that enabled emergency departments to have access to patient records held by GPs in primary care and vice versa.

*“I think the issues that we’re going to have is sharing information. Both in terms of data sharing rules, but also politics, culture interest. For example, within our local authority, they’ve got a community engagement manager who knows the addresses of each gypsy Roma traveller place. But they’re on private land and she’s not allowed to share that information with us. But we need to provide healthcare to those people. The only way that information can be shared is to take us onto those sites and introduce us to those communities. And then get their permission to come back and provide healthcare.”* [Transformation Lead]

### Law 2: Integration costs before it pays

Perceptions about the costs of setting up and running an ICS and how funding should be allocated, were important concerns for interviewees.

Interviews and meeting observations indicated that substantial financial and human resource were needed to develop system building blocks such as new governance arrangements and infrastructure development. The four ICS had variable existing capacity to draw upon, and therefore had very different starting points. For example, in terms of quality improvement (QI) (data analytics and QI expertise), there was substantial variation in existing capacity across the ICS. Some ICSs mentioned plans to create QI training hubs and academies for ICS staff thereby boosting QI capacity.

It was recognised that without developing the digital infrastructure to enable data integration, several ICS goals would not be met. However, this would require huge investment against the backdrop of a challenging economic climate, which led to scepticism about whether the funding would be made available. Some suggested that the timing of this major reorganisation posed challenges and that funds might be better allocated to other areas of greater need such as backlogs and workforce recruitment and retention.

*“But we need a massive investment in our digital infrastructure and also a recognition that even if that money arrived today we’re not going to see the benefits for a couple of years because of the scale of the digital change, it’s just massive, it’s going to take such a long time.”* [Chief Nurse]

Tensions around the allocation of funding were also observed. Individual local authorities were keen to ensure they continued to receive the same funding as before, or more. Some interviewees expressed concerns that acute care providers, now joining forces into provider collaboratives, may demand a higher share which could lead to an inequitable distribution of funding, risking disengagement from the primary and social care sectors.

*“I struggle to see how the money allocation is going to work, I know there’s supposed to be one pool and we have to be a lot more altruistic about how it’s all shared. And I’m all for that, but the reality will be different and if we say that 80% of care happens in the community, the local authority rather than health, that has implications. Well it’s the same with general practice, you know, 80% of care happens in general practice, with a small percentage in specialist care but that takes all the money.” [Clinical Commissioning Group- CCG Chair]*

### Law 3: Your integration is my fragmentation

Interviewees mentioned that some of the integrated care initiatives planned by ICSs could result in fragmentation of services, due to unnecessary duplication, differences in perspective, culture and priority setting, and changes of structures.

Some interviewees, especially from local authorities described how ICSs were duplicating certain functions which already existed. For example, it was felt that housing which falls under the local authority remit at place did not require additional strategic consideration from the ICS. These duplications created unease between system and place and between the ICS and local authorities who felt undermined.

Some interviewees raised the key differences in the organisational/professional culture between health and social care. For example, a ‘top down and bureaucratic approach’ of the NHS was seen by some as a barrier to integration. Furthermore, the well-established differences in professional culture such as the risk averse, biomedical model practiced by health professionals compared to the person-centred, function optimisation approach of care professionals was seen as a hindrance to integration at the service delivery level.

There were also some tensions arising when priorities set at system level did not match with local priorities. Interviewees described a lack of understanding from system leaders as to how to best balance system and place-based efforts and approaches. That said, one ICS consulted with each of its places at length to ensure that system priorities reflected individual place priorities.

Not all partners were supportive of integration, and some were reluctant to commit to partnership working. This was most evident in ICS where local authorities were represented by different political parties. Where these political differences existed, local authorities were more committed to place-based working and less interested in system level collaboration.

*“I think something that I personally feel could be difficult is that, particularly the NHS, but the council too is obviously there’s a big political drive or influence around it and scrutiny. And also, the differentiation because the NHS is still fairly centralised command and control type structures, it’s trying to move away from that. But it still is, ‘you will do this’ and you have no choice, and it changes on a whim a little bit. Whereas councils as I understand them, it’s localised, and actually the local lead of the council is that authority and sets the tone and I wonder if that’s a tension or could be a tension.”* [Business Development Manager]

The reorganisation of structures to form an ICS/ICB has created uncertainty for staff at senior and middle management levels. This has led to workforce attrition in managerial roles with an associated loss of experience and knowledge while also creating difficulties in recruiting to the new roles required for the ICS. Additionally, the reorganisation of commissioning has led to a reduced role for primary care in funding and resource allocation, leading to concerns that primary care may be less engaged in integration at the system level.

### Law 4: You can’t integrate a square peg into a round hole

Interviewees focussed less on the key premise of this law i.e. shifting care away from acute settings to the community and more on other key goals of the ICS (how to improve whole population health, prevention and reducing inequalities) and holding both system and place to account in delivering these goals. There was a changing emphasis from focusing on treating illness, to a greater focus on how to improve population health. Issues around accountability for health and care organisations were causing tensions, as were issues around what should be done at place level, and at system level.

The interview data indicated a substantial emphasis on improving population health by addressing wider social determinants, with the local authority and specifically public health directorates playing a more active role in strategy and decision-making. It was clear from the interviews and meeting observations that inequalities were at the forefront of senior leaders’ priorities although this was tempered somewhat by an underlying concern that the extensive pressures on health and care systems in the short-medium term could supplant a wider population health focus. There was also a sense that the established focus of some NHS commissioners and providers on illness had been retained. For example, acute trusts were less engaged in population health and did not necessarily perceive it as a priority for their day-day work. One notable exception was discussions around taking a clinical approach using an inequalities lens to address lengthening to elective lists

*“..in terms of how we manage our elective waiting lists we take into consideration and we look and we do data trawls in terms of the ethnicity of people on our waiting lists and where there is disproportionate waiting to certain groups of individuals. So yes, absolutely, it is part of our thinking now in terms of how we deliver the current services but also how we are thinking about the delivery of services in the future.”* [Acute Trust CEO]

There were clear differences relating to accountability between health and social care organisations. Interviewees believed that NHS organisations were not just accountable to themselves but also to ICBs through shared accountability and of course through their statutory obligations to NHS England (as the arbiter of the NHS). In contrast, local authorities are accountable to the local population as councillors are elected individuals. Interviewees mentioned that the notion of democratic accountability was unfamiliar to the NHS hence they struggled to grasp this aspect when developing and implementing the ICS strategy which, in turn, had implications for partnership working.

‘*Local authorities have been working with local people forever, since they’ve existed, whereas the NHS sometimes, because it has that sort of more national direction set, it can often feel like it looks up to the centre rather than out to its population. Yeah, like I say, the opposite is true for local authorities, who have that kind of democratic accountability, so therefore have that engagement and understanding of their communities much more.’* [ICB executive member]

At senior levels there was acknowledgment of the principal of subsidiarity. ICS were still trying to understand what was best done at place level, and where the system could add value. Some interviewees expressed concerns that ICS were determining what needed to be done at place without engaging place or utilising local assets and partners. In general, local authority interviewees didn’t see the value of working at system level and wanted greater devolution to place and more autonomy, particularly over decision making and expenditure.

### Law 5: The One Who Integrates Calls the Tune

Across interviewees there was a strong emphasis on ICSs working in partnership, across health, social and voluntary care, with representatives of local people, the public and communities and with external agencies. This would require NHS organisations to relinquish some control over matters pertaining to governance and strategic decision making. Co-production had the potential to enable greater person and public involvement but there were important factors that would hinder parity for non-health partners. There were issues around unequal partnerships, with the NHS organisations dominating the agenda, and mixed views on the role of NHS England’s top-down approach.

Co-production was viewed as an effective mechanism to involve the public and local communities, however, misconceptions existed on its meaning, how it should be practiced in a meaningful way and who should be responsible for this. There was a reliance on HealthWatch as a single voice of patients given their access to insights and experiences of local people using health and care services. However, HealthWatch felt that the insights they could share with the ICS were not entirely representative of local people and with limited capacity and resources,they suggested they may struggle to expand their work in line with ICS ambitions. Moreover, several participants felt that if ICS were to succeed in their goals of improving population health and addressing inequalities, they would have to involve communities and local people at every stage of development and build bottom up.

*“I think that one of the things that we’re saying to everyone is, you know, if you want to have a devolved budget and you want to be your own commissioner, then we need to see you building your services around co-production. So that’s one of our sort of fundamental values. I think that one of the things we need to do is get serious about that. And get beyond – you know, Healthwatch are a useful organisation, they’re a useful conduit into people, but they’re not the views of everyone*. [ICS CEO]

Despite a consensus among health partners for more partnership working and local authorities and HealthWatch suggesting that working relationships with the NHS were gradually improving, ICSs were struggling to dispel the preponderance for the NHS with the agenda dominated by elective waiting lists and the demands on emergency and primary care. Hence, neither local authorities nor Healthwatch considered themselves as equal partners despite their membership on the ICB and multiple other ICS committees.

Participants’ views on the role of external agencies, particularly guidance from NHS England (NHSE) was mixed. While some found it helpful in relation to setting up structures, some felt it was too prescriptive and imposed unrealistic timeframes. Some perceived NHSE as imposing an assurance-based focus on performance metrics related to current pressures on the system in primary and acute care. Given the predominance of CCG staff in ICS’s who are accustomed to contracts-based management and assurance, there were concerns that the wider goals of population health improvement and inequalities could be impeded in the short term.

*‘..what I am predicting will happen is that the Integrated Care Systems will become overly assurance focussed because that’s what they feel that they have to do to meet the needs of the national team. They will be overly focussed on risk and areas where people are struggling.’* [Chief Quality Officer]

## Discussion

This study presents an early overview of how ICSs in England are developing through an analysis of four case sites. The findings identify the benefits of ICSs such as their potential in enhancing the delivery of good quality care, improving population health and providing more person-centred treatment and care in the community. However, previously cited longstanding challenges for integrated care are also evident in this study [2,4,11]. This includes differences between health and social care in terms of accountability and organisational/professional cultures and between the key priorities of system versus those of place. Moreover, issues of data integration, struggles in engaging local communities in ICS development, risks of duplicating efforts and tensions over funding allocation threaten to hamper integration.

While the study is focussed on England, the findings hold salience for other countries embarking on a whole systems approach to integration while also providing important learning for integrated care more broadly. Targeting efforts to integrate care on interdependent areas of health and care i.e. linkages (services and pathways) or coordination (integrated service delivery) as opposed to full integration warrants further consideration. It has been suggested that as systems and services become more integrated, the need for coordination diminishes [27]. In contrast, our findings suggest that full (structural) integration of infrastructure (e.g. digital data integration), governance and management systems is resource intensive and may be contentious in terms of resource allocation. This poses a conundrum for policymakers about how to best allocate and optimise resources while they grapple with a rising demand for healthcare amid increasing economic uncertainty.

A further consideration for integrated care systems globally is how to better manage population health and wellbeing. We identified a concerted willingness among all providers to address this issue with an ambition to improve wellness and reduce disease. This is in stark contrast to the early goals of integrated care initiatives, especially in England which prioritised care coordination for the cohort of the population with the most complex care needs [1]. In other countries, improving population health has been a key goal of integrated care systems for some time, recognising that without a prevention strategy there may come a point where demand for care will not just surpass supply but overwhelm services [28]. Some ponder whether we have already reached that tipping point in England and hence, policymakers, regulators and system leaders maybe be distracted by the system pressures in primary care, emergency departments and hospital discharge to the detriment of improving population health [29].

We noted some promising signs that local authority and NHS providers were keen to collaborate and invest in partnership working across the system, but the goals of English ICSs expect systems to go further and strengthen the relational aspects of integration by engaging local people and communities in co-production. Studies of co-production in the context of integrating health and social care underline the contention and diversity in the understanding of the term; how unhelpful the complexities of organisations are in facilitating co-production and how essential resources are to underpinning its development [30]. These issues are evident in this study and are compounded by an overreliance on bodies such as Healthwatch to provide insights to inform ICSs of patients’ views, which is not a sustainable policy. As demand grows, Healthwatch’s capacity to fulfil this role will be limited. A recent review of co-production approaches in the context of service integration argues for the need for a ‘pragmatic’ and realistic model of co-production that works within the roles and constraints of professionals and providers [31].

This study has demonstrated *Leutz’s framework* [16] remains relevant in the context of ICS in England over two decades after its initial publication: many of the challenges described by Leutz apply specifically to the set-up of ICSs, and may be relevant for the set-up of integrated care initiatives elsewhere. We did find some of the laws more challenging to apply as they lacked relevance in the context of the plethora of system reorganisations in England over the last 20 years. There was also an added complexity to applying the original framework as the examples exemplifying the laws from both the US and the UK represent two vastly different healthcare systems in terms of their funding model and organisational structures, although there are some commonalities between the current guise of English ICS and ACOs in the US.

Learning from earlier evaluations of integrated care initiatives in England (albeit at sub-regional or place level) about the challenges of integration remain relevant in the context of ICS. *Lewis et al* suggest that integration is not a short term endeavour and hence results from evaluation conducted over short periods of time will indicate modest impact of integrated care initiatives best illustrated by a moderate reduction in unplanned hospital admissions which is often the primary measure of the success of integration [11]. Hence, given this study was undertaken during the early stages of ICS formation we suggest that further research and evaluation of ICS in England are undertaken at regular intervals over the coming years. ICSs need time to set up the basic building blocks and form the necessary relationships across the system before they can really start tackling some of their wider goals of improving population health and reducing inequalities. We therefore suggest that research focusses on whether ICS are meeting their wider goals in reducing inequalities and engaging local people and communities as well as on outcomes that indicate whether ICS are delivering more person-centred care.

### Strengths and Limitations

A possible limitation of this study is that the primary focus of the broader project was on the management and assurance of quality. That said, the breadth and depth of our interviewee sample and the topics covered during interviews meant we garnered a lot of information about the development of ICSs more generally. Indeed, the diversity of our interview sample is a key strength of this study as we purposively selected representatives of non-health provider organisations to obtain a broad perspective of views on ICSs.

### Lessons learned from this study

Despite the development of ICS, the independence of NHS and local authorities has been retained because of distinct models of financing from central government, differing accountability mechanisms (NHSE vs local population) and contrasting organisational and professional cultures. Unsurprisingly, this has led to concerns about the parity of esteem between health and social care organisations, which has implications for partnership working. Multi-sector and multi-professional committees and Boards can support collaboration, but they are not a panacea. As we have seen with multi-professional teams in the community sector, even co-terminosity does not guarantee effective partnership working [32].

The centralisation of healthcare in England is in stark contrast to similar countries in Europe and elsewhere, where devolved, localised and bottom-up models governance of dominate [33,34]. NHSE continues to impose, prescribe and direct – best illustrated by their unrelenting focus on quality assurance and performance-based metrics which may detract from prevention and improving population health. For ICS to truly pursue local approaches to managing local problems, they will require greater autonomy in determining how they measure success i.e. creating metrics to measure locally relevant outcomes that extend beyond the arbitrary measures of performance such as non-elective attendances or A&E wait times and instead focus on addressing health inequalities.

The ICS approach to population level planning is both innovative and ambitious and as such, is likely to be fraught with enduring challenges unless certain steps are taken. This includes joint working with local communities through co-production to ensure that services are tailored to the needs of the local population and greater investment in digital tools such as shared electronic records to ensure that the patient/user journey across care pathways is seamless and safe.

## Conclusion

The formal launch of ICSs in England signalled a significant shift in the organisation and delivery of health and social care with a considerable scale of ambition. The remit of ICSs has expanded to managing the health and care of the wider population which represents a seismic policy change. The unprecedented pressures on health and care systems to focus on key operational issues such as reducing demand on primary care, tackling the post Covid elective backlog and managing urgent and emergency care may draw attention away from improving population health and prevention. Underpinning factors such as relationships across system partners and community and public involvement will struggle to flourish under these circumstances. In the context of external and internal pressures, policymakers and regulators are likely to become more risk adverse and hence more insular reverting to focussing on their own organisation with a reluctance to commit resources to system wide working to the detriment of integration efforts. In this climate, it is questionable how ICS can achieve their aims of reducing inequalities and a greater focus on prevention while simultaneously building the digital infrastructure, staff expertise and culture to support such a shift.
